# Anti-Inflammatory Versus Antifibrotic Therapies for the Management of Rheumatoid Arthritis–Associated Interstitial Lung Disease: Protocol for a Systematic Review and Meta-Analysis

**DOI:** 10.2196/73219

**Published:** 2025-07-21

**Authors:** Sneh Sonaiya, Alexandra Jianu, Nicholas Jianu, Kavita Batra

**Affiliations:** 1 Department of Internal Medicine University of Nevada, Las Vegas Las Vegas, NV United States; 2 Department of Medicine Lake Erie College of Osteopathic Medicine Erie, PA United States

**Keywords:** Rheumatoid arthritis, interstitial lung disease, anti-inflammatory, antifibrotic, autoimmune, pulmonary fibrosis

## Abstract

**Background:**

Rheumatoid arthritis (RA) is a chronic autoimmune inflammatory disease that affects approximately 0.5% to 1% of the population in the United States and Northern Europe. Interstitial lung disease (ILD) is the most common and severe pulmonary manifestation of RA, collectively referred to as RA-associated ILD (RA-ILD). RA-ILD contributes significantly to morbidity and mortality and often presents with a variable clinical course. Although corticosteroids and disease-modifying antirheumatic drugs (DMARDs) remain the cornerstone of RA management, their role in RA-ILD is less clearly defined. In contrast, antifibrotic therapies such as pirfenidone and nintedanib, initially developed for idiopathic pulmonary fibrosis, are now being explored for their potential in treating fibrosing variants of RA-ILD. Despite increasing clinical use, no systematic review has comprehensively compared the safety and efficacy of antifibrotic versus anti-inflammatory therapies in chronic RA-ILD.

**Objective:**

This study aims to compare the impact of antifibrotic and anti-inflammatory therapies on lung function, radiologic progression, clinical outcomes, and safety in patients with chronic RA-ILD.

**Methods:**

This study will follow PRISMA (Preferred Reporting Items for Systematic Reviews and Meta-Analyses) guidelines and is registered with PROSPERO (CRD42024583847). A comprehensive search of PubMed, Embase, and the Cochrane Library will be conducted for studies published between January 1991 and August 2024. Eligible studies will include adult patients (aged ≥18 years) with a diagnosis of RA and confirmed ILD based on radiological or histopathological findings who have been treated with either antifibrotic or anti-inflammatory therapies. The PECOS (Population, Exposure, Comparator, Outcome, Study Design) framework will be used to define inclusion criteria. The primary outcomes assessed in this review will include the following pulmonary function parameters: forced vital capacity, forced expiratory volume in 1 second, and diffusing capacity of the lungs for carbon monoxide. Anti-inflammatory therapies will be stratified into corticosteroids, conventional synthetic DMARDs, and biologic DMARDs to account for heterogeneity. The Cochrane Risk of Bias 2 (RoB 2) and Risk of Bias in Non-randomized Studies - of Interventions (ROBINS-I) tools will be used for quality assessment, and GRADE (Grading of Recommendations Assessment, Development, and Evaluation) methodology will be used to evaluate the certainty of evidence.

**Results:**

The literature search and screening commenced in August 2024, and data extraction is underway. The final results are expected by December 2025.

**Conclusions:**

This systematic review and meta-analysis will provide a comprehensive comparison of antifibrotic and anti-inflammatory therapies in the treatment of chronic RA-ILD. The findings will help inform clinical decision-making, support evidence-based treatment selection, and identify gaps in current research. By addressing both efficacy and safety, this review aims to guide future studies and improve patient outcomes for this complex and debilitating condition.

**Trial Registration:**

PROSPERO CRD42024583847; https://www.crd.york.ac.uk/PROSPERO/view/CRD42024583847

**International Registered Report Identifier (IRRID):**

DERR1-10.2196/73219

## Introduction

Rheumatoid arthritis (RA) is a prevalent autoimmune inflammatory condition affecting approximately 0.5% to 1% of the population in the United States and Northern Europe [1]. Interstitial lung disease (ILD) is the most common respiratory manifestation of RA and can lead to significant morbidity and increased mortality in some patients with RA-associated ILD (RA-ILD). The prevalence of RA-ILD among individuals with RA ranges from 2% to 8% [2]. RA-ILD often follows a clinically unpredictable course, with recent studies reporting an increasing prevalence of RA-ILD ranging from 27% to 67%, a notable proportion of which includes asymptomatic patients [3].

RA-ILD is believed to result from persistent immune activation and inflammation, which leads to excessive fibroproliferation in the lung tissue of individuals with a genetic predisposition [4]. In some cases, the clinical symptoms of ILD may appear before the onset of articular symptoms in patients with RA. One study found that approximately 14% of patients with RA-ILD were diagnosed with ILD 1 to 5 years prior to being diagnosed with RA [5]. Several risk factors for the development of ILD in patients with RA have been identified, including older age, male sex, cigarette smoking, and the presence of positive anticyclic citrullinated peptide antibodies or immunoglobulin M rheumatoid factor [6-9].

RA-ILD can be classified into acute, subacute, and chronic forms based on the clinical presentation. In patients with acute RA-ILD, characterized by diffuse alveolar damage, high-dose corticosteroids are typically the first-line treatment. About half of patients with RA develop ILD either before or within 5 years of the RA diagnosis [10]. In contrast, the management of chronic RA-ILD focuses on controlling the underlying disease to prevent further progression of ILD. Disease-modifying antirheumatic drugs (DMARDs) have traditionally been used to manage RA-associated symptoms. For RA-ILD specifically, therapies such as corticosteroids, mycophenolate, azathioprine, rituximab, and TNF-α inhibitors are commonly used. Antifibrotic therapies, including pirfenidone and nintedanib, have been approved by the United States Food and Drug Administration for idiopathic pulmonary fibrosis and are now being explored for RA-ILD [11].

Several clinical trials have demonstrated the safety and efficacy of antifibrotic agents in the treatment of ILD [12-14]. However, a comprehensive review comparing the safety and efficacy of antifibrotic versus anti-inflammatory therapies in patients with chronic RA-ILD remains lacking. In this systematic review and meta-analysis, we aim to compare the safety and efficacy of these 2 therapeutic approaches in the management of chronic RA-ILD. By examining the comparative benefits and limitations of these treatment strategies, this study aims to provide valuable insights to guide clinical decision-making and improve the management of patients with chronic RA-ILD.

## Methods

### Ethical Considerations and Guidelines

As this study does not include the direct involvement of human participants, an institutional ethical review will not be warranted. However, to adhere to a strict methodology, the protocol of this systematic review was registered on PROSPERO (CRD42024583847) [15]. PROSPERO is an international database of prospectively registered systematic reviews, which provides a unique permanent registration number to the protocol that prevents duplication, thereby reducing reporting bias. We followed the PRISMA (Preferred Reporting Items for Systematic Reviews and Meta-Analyses) guidelines in conducting and reporting this systematic review to ensure a comprehensive and transparent approach to the evaluation of the available evidence (Multimedia Appendix 1) [16].

### Review Interest

The study will analyze the effectiveness of antifibrotic and anti-inflammatory therapies in improving or stabilizing lung function and clinical outcomes in adult patients with RA-ILD.

### Inclusion and Exclusion Criteria

The inclusion criteria for this systematic review were developed using the PECOS (Population, Exposure, Comparator, Outcome, Study Design) framework to ensure that only studies directly relevant to the research question are included (Table 1) [17]. Eligible studies will include adult patients (aged ≥18 years) with a diagnosis of RA and confirmed ILD based on radiological or histopathological findings who have been treated with either antifibrotic or anti-inflammatory therapies. The primary outcomes assessed in this review will include the following pulmonary function parameters: forced vital capacity (FVC), forced expiratory volume in one second (FEV1), and diffusing capacity of the lungs for carbon monoxide (DLCO). Secondary outcomes may include radiological changes in high-resolution computed tomography (HRCT), clinical end points (eg, overall survival), and safety and tolerability of treatment. We will include randomized and nonrandomized clinical trials, case-control studies, and prospective or retrospective cohort studies. Only studies involving human patients and adult populations and published in English between January 1, 1991, and August 1, 2024, will be considered, regardless of geographic location. Exclusion criteria include studies involving nonhuman subjects, pediatric populations, or other ILD subtypes not specifically related to RA. Studies that do not evaluate antifibrotic or anti-inflammatory therapies or that fail to report quantitative data on lung function or clinical outcomes will be excluded. Additionally, case reports, abstract-only publications, review articles, editorials, short communications, and opinion pieces will be excluded. The decision to limit inclusion to English-language publications was made due to resource constraints and the lack of access to reliable translation services, which may impact the feasibility and consistency of non-English data extraction and interpretation. Furthermore, gray literature such as conference abstracts, dissertations, and preprints were not included in this review due to concerns about inconsistent reporting quality and the inability to assess methodological rigor or extract full datasets. In case of missing data, we will assess the potential impact through sensitivity analyses and report the extent of missing data in our results.

**Table 1:** PECOS (Population, Exposure, Comparator, Outcome, Study Design) criteria for the systematic review of rheumatoid arthritis-associated interstitial lung disease therapies.

PECOS criteria	Details
Population	Adult patients (aged ≥18 years) diagnosed with rheumatoid arthritis (RA) and confirmed interstitial lung disease (ILD) via radiological or histopathological findings.
Exposure	Antifibrotic therapy for the treatment of RA-associated ILD.
Comparator	Anti-inflammatory therapy for the treatment of RA-associated ILD.
Outcomes	Change in lung function parameters (eg, forced vital capacity, forced expiratory volume in 1 second, and diffusing capacity for carbon monoxide), and changes observed on high-resolution computed tomography.
Study design	Included: Studies that compare the efficacy of antifibrotic versus anti-inflammatory therapies, including randomized controlled trials, cohort studies, and observational studies. Excluded: Case studies, case reports, abstract-only studies, reviews, editorials, short communication, and opinion articles.

### Informational Sources and Search Strategy

A comprehensive search strategy was developed by the study investigators, with guidance from a librarian, to identify relevant articles across multiple databases, including PubMed, Embase, and Cochrane Library. The search was designed to capture studies related to RA-ILD and treatment options involving antifibrotic or anti-inflammatory therapies. The search strategy was refined through preliminary searches in Embase, with additional terms introduced as new concepts were identified. Keywords included combinations of terms such as “rheumatoid arthritis,” “interstitial lung disease,” “antifibrotic therapy,” “anti-inflammatory therapy,” “pirfenidone,” “nintedanib,” “disease-modifying anti-rheumatic drugs (DMARDs),” and “high-resolution computed tomography (HRCT).” These terms were selected to ensure comprehensive coverage of studies addressing the relevant interventions and outcomes in RA-ILD. The search strategy in detail is highlighted in detail in Multimedia Appendix 2.

### Screening

All articles will be imported into an intelligent systematic review tool, Rayyan (Rayyan Systems Inc), for screening. After deduplication, the records will be independently reviewed by 2 reviewers to evaluate eligibility based on predefined inclusion and exclusion criteria. Screening will be conducted systematically and sequentially, starting with titles, followed by abstracts, and then full-text assessments. Reasons for excluding studies will be documented at each step of the screening process to ensure transparency. The study selection process will be visually represented using a PRISMA flow diagram.

### Data Extraction and Main Data Elements

Two reviewers will independently extract the relevant data elements from the eligible full texts of the articles and will record these variables in a standardized code book. A double extraction method will be used to ensure accuracy and completeness. After data extraction, disparities will be resolved by consensus and discussion with the third reviewer or a “tiebreaker.” Attempts to contact the corresponding authors of the included articles will be made if more information about the individual study data is needed. The following data elements will be extracted: study title, authors, year of publication, study design, quality score, total sample size, sex distribution (percentage of male to female patients), mean age and SD, duration of RA diagnosis (mean or median and SD), duration of ILD (mean or median and SD), geographic location (country and region if applicable), data on comorbidities (eg, cardiovascular disease, diabetes, and smoking history), baseline lung function parameters (eg, FVC, FEV1, and DLCO with units and baseline values), names of the antifibrotic drugs used (eg, nintedanib and pirfenidone), names of the anti-inflammatory drugs used (eg, methotrexate and TNF inhibitors), dosage regimen, treatment duration, adverse events related to therapies (eg, side effects of antifibrotic or anti-inflammatory treatments), qualitative changes in fibrosis patterns (eg, ground-glass opacities and honeycombing), effect sizes for outcomes (eg, mean differences, hazard ratios, and odds ratios), CIs and *P* values, clinical endpoints (overall survival and progression-free survival), and the type of anti-inflammatory treatment. To improve comparability across studies and reduce heterogeneity, anti-inflammatory therapies will be categorized into subgroups based on pharmacologic class, including systemic corticosteroids, conventional synthetic DMARDs (eg, methotrexate and azathioprine), and biologic DMARDs (eg, TNF inhibitors and rituximab). These subgroups will be analyzed separately where the data permit, in accordance with classifications used in prior RA-ILD studies.

The primary outcomes of this study will focus on pulmonary function parameters to evaluate the impact of interventions on lung function. These include FVC, FEV1, and DLCO. For each parameter, both absolute values and percentages of predicted normal will be assessed, alongside changes from baseline to follow-up.

The secondary outcomes will focus on radiological changes evaluated using qualitative changes in high-resolution computed tomography (HRCT) to assess fibrosis patterns, including ground-glass opacities and honeycombing, and to determine the progression, stabilization, or regression of fibrosis. Additionally, clinical end points such as overall survival, measured as the time from baseline to death from any cause, will be studied. Lastly, safety and tolerability will be assessed by monitoring adverse events related to the therapies used in the study. The key primary and secondary outcomes of the study are highlighted in Table 2. For outcomes such as disease progression, regression on HRCT, and exacerbations, we will use the definitions as provided by each individual study. No single standardized definition will be applied across studies, but variations in definitions will be documented and considered in the interpretation of the results. Where feasible, subgroup or sensitivity analyses will be conducted to account for heterogeneity in outcome definitions.

**Table 2:** Key primary and secondary outcomes of the study.

Outcome			Description
**Primary outcomes**			
	Pulmonary function parameters	Forced vital capacity, forced expiratory volume in 1 second, and diffusing capacity for carbon monoxide	
**Secondary outcomes**			
	Radiological changes	Fibrosis regression, stabilization, or progression evaluated through high-resolution computed tomography scans	
	Clinical end points	Disease progression rates, frequency of exacerbations, overall survival, and quality of life measures	
	Safety and tolerability	Adverse events related to the therapies	

### Quality or Risk of Bias Assessment

Risk of bias for each included study will be independently assessed by 2 reviewers. For randomized controlled trials, we will use both the Cochrane Risk of Bias 2 (RoB 2) tool and the National Heart, Lung, and Blood Institute quality assessment tool [18,19]. Two independent reviewers will evaluate the full texts and score them separately. Inter-rater agreement will be measured using κ statistics. The National Heart, Lung, and Blood Institute tool consists of 14 checklist items that cover all key components of original research studies. Based on the tool’s guidelines, the quality will be rated as poor (0-4 out of 14), fair (5-10 out of 14), or good (11-14 out of 14).

For nonrandomized studies, we will use the Risk Of Bias In Non-randomized Studies – of Interventions (ROBINS-I) tool, which assesses bias across domains such as confounding, selection of participants, classification of interventions, and measurement of outcomes [20]. Discrepancies in assessments will be resolved through discussion or consultation with a third reviewer. All risk of bias evaluations will inform the interpretation of the results and contribute to the GRADE (Grading of Recommendations Assessment, Development, and Evaluation) assessment of the overall quality of evidence.

### Statistical Plan

The initial results of all the studies ultimately included in the analysis will be summarized in a concise table. The pooled effect estimates, along with their 95% CIs, will be calculated using Comprehensive Meta-Analysis software (version 3.0; Biostat). Given the expected heterogeneity across the included studies, a random-effects model will be used to compute the pooled estimates, as it provides a more robust approach by accounting for variability between studies.

To assess the overall quality and strength of the body of evidence for each key outcome, we will apply the GRADE approach. The quality of evidence will be rated as high, moderate, low, or very low based on factors such as study limitations, inconsistency, indirectness, imprecision, and publication bias. GRADE assessments will be performed independently by 2 reviewers, with discrepancies resolved through consensus or consultation with a third reviewer.

Furthermore, to evaluate heterogeneity, both the Cochran *Q* test and the *I*² statistic will be applied. These measures will help determine the extent of variability that is due to true differences across studies, rather than being attributable to random chance. The Cochran Q test will assess the overall heterogeneity, while the *I*² statistic will quantify the proportion of total variation across studies that is due to differences in study outcomes.

If a sufficient number of studies are available, subgroup analyses will be performed to examine the influence of moderator variables, such as sociodemographic factors, country, and types of interventions, on the outcomes. Additionally, sensitivity analyses will be conducted to determine if any individual studies have an outsized effect on the pooled estimates.

To assess publication bias, a funnel plot will be created, and an Egger linear regression test will be conducted. The significance level for all tests will be set at a 2-sided *P* value of <.05. In the meta-analysis, effect sizes will be presented using standardized mean differences for continuous outcomes and risk ratios for dichotomous outcomes. Standardized mean differences will allow for the comparison of effect sizes across studies with different measurement scales, while risk ratios will quantify the likelihood of an outcome occurring in the treatment group relative to the control group. Forest plots will be used to visually represent the effect estimates and their associated CIs, providing a clear depiction of the pooled results.

## Results

The literature search and screening commenced in August 2024 and are projected to be completed by August 2025. We are currently in the data extraction phase of this systematic review and meta-analysis. A detailed overview of the project timeline and key milestones is shown in Table 3. No external funding was obtained for this project. The final results are anticipated to be submitted for publication by December 2025.

**Table 3:** Timeline for systematic review and meta-analysis tasks.

Tasks	Aug 2024	Sep 2024	Oct 2024	Nov 2024	Dec 2024	Jan 2025	Feb 2025	Mar 2025	Apr 2025	May 2025	Jun 2025	Jul 2025	Aug 2025
Initial design and searches	✓	✓											
Screening of duplicates and titles			✓	✓									
Screening of abstracts					✓	✓							
Screening of full-text articles							✓	✓					
Search references of included studies									✓				
Data extraction										✓	✓		
Synthesis and risk of bias assessment											✓	✓	
Analysis												✓	
Abstract and manuscript drafting													✓
Submission to conferences and peer-reviewed journals													✓

## Discussion

### Overview

RA-ILD represents a complex and challenging aspect of managing patients with RA. This systematic review and meta-analysis aims to comprehensively assess the efficacy of antifibrotic and anti-inflammatory therapies in patients with chronic RA-ILD, highlighting the comparative benefits and limitations of these therapeutic strategies. The prevalence of RA-ILD is significant, and its clinical presentation varies widely. Given the complexity and heterogeneity of RA-ILD, it is imperative to tailor treatment strategies based on the underlying pathophysiology and patient characteristics.

### Diagnostic Imaging in RA-ILD

RA-ILD can be diagnosed based on histopathologic and imaging features. The most common subtype of RA-ILD is usual interstitial pneumonia (UIP), which is characterized by fibrosis and is associated with poor prognosis. There is growing evidence to support differentiating UIP from non-UIP patterns in patients with RA-ILD due to differences in clinical outcomes [21,22]. The prognosis of patients with RA-ILD with UIP patterns is poorer than that of patients with non-UIP patterns [23]. Patients with RA-ILD with UIP tend to be older individuals and seem to be less responsive to conventional treatment compared to patients without UIP [24,25]. HRCT serves as a reliable tool for accurate identification of the histopathologic UIP pattern, with a positive predictive value of 95% (19 out of 20 cases) [26]. The presence of definite HRCT UIP patterns characterized by basal and subpleural interstitial reticulations, fibrosis, traction bronchiectasis, and honeycombing exhibits a specificity of 96% (26 out of 27 cases) and a sensitivity of 45% (19 out of 42 cases) in relation to histopathologic UIP patterns [26]. Nonspecific interstitial pneumonia is also common in some patients with RA-ILD. Nonspecific interstitial pneumonia is characterized by parenchymal inflammation and has a better prognosis compared to UIP. Other less common RA-ILD subtypes include lymphocytic interstitial pneumonia, acute interstitial pneumonia, organizing pneumonia, desquamative interstitial pneumonia, and respiratory bronchiolitis ILD [27]. In most cases, radiographic imaging in the form of HRCT is sufficient to identify the underlying fibrosis pattern, and surgical biopsy is reserved only for patients in whom radiographic imaging is not diagnostic [28].

### Strengths and Limitations

This systematic review has several strengths, including a comprehensive evaluation of the literature and rigorous methods to assess study quality and minimize biases. However, limitations exist. One major challenge is the heterogeneity among the studies, stemming from differences in methodologies, settings, interventions, diets, and outcome measures, which may impact the consistency of the findings. Reviewer bias is another concern, but we will address this by using standardized risk of bias tools for each study and conducting sensitivity analyses to assess the impact of low-quality studies on the overall results. To further ensure objectivity, we will seek input from subject matter experts and maintain transparency throughout the process. Furthermore, anti-inflammatory agents are often prescribed primarily for articular RA control rather than for the treatment of RA-ILD itself, whereas antifibrotic drugs are typically initiated specifically for ILD. This potential misclassification of therapeutic intent could confound comparative-effectiveness results.

Another limitation is the potential for missing relevant studies despite a broad search strategy developed with the help of a medical librarian. This risk could affect the completeness of the review. Additionally, publication bias may skew the results, as studies with positive outcomes are more likely to be published. To address this, we will assess publication bias through funnel plots and Egger regression tests, helping to ensure a balanced and accurate interpretation of the evidence.

### Dissemination Plan

The findings from this systematic review will be disseminated through a peer-reviewed journal article and presentations at academic conferences.

### Conclusions

This study will provide a comprehensive review comparing antifibrotic and anti-inflammatory therapies in the management of chronic RA-ILD. This study will include data on treatment efficacy, patient outcomes, radiologic progression, symptom burden, and quality of life for patients with RA-ILD following antifibrotic and anti-inflammatory therapies, thereby offering insights for the management of chronic RA-ILD.

## Appendix

Multimedia Appendix 1 PRISMA-P (Preferred Reporting Items for Systematic Reviews and Meta-Analysis Protocols) checklist.

Multimedia Appendix 2 Research query for database search.

## Abbreviations

DMARD: disease-modifying antirheumatic drug
DLCO: diffusing capacity of the lungs for carbon monoxide
FEV1: forced expiratory volume in 1 second
FVC: forced vital capacity
GRADE: Grading of Recommendations Assessment, Development, and Evaluation
HRCT: high-resolution computed tomography
ILD: interstitial lung disease
PECOS: Population, Exposure, Comparator, Outcome, Study Design
PRISMA: Preferred Reporting Items for Systematic Reviews and Meta-Analyses
PROSPERO: International Prospective Register of Systematic Reviews
RA: rheumatoid arthritis
RA-ILD: rheumatoid arthritis-associated interstitial lung disease
UIP: usual interstitial pneumonia

## Data Availability

The data analyzed in the study will be available upon request from the corresponding author.
