# Supplementary material for: Anti-Inflammatory Versus Antifibrotic Therapies for the Management of Rheumatoid Arthritis–Associated Interstitial Lung Disease: Protocol for a Systematic Review and Meta-Analysis
Source: JMIR Res Protoc. 2025 Jul 21;14:e73219. doi: 10.2196/73219 (PMC12322608; doi:10.2196/73219)
Supplement: Multimedia Appendix 2 [file resprot_v14i1e73219_app2.docx]

| **Database** | **Query Used** | **Limit Criteria** | **Number of Results** |
| --- | --- | --- | --- |
| **PubMed** | (("Arthritis, Rheumatoid"[MeSH] OR "Rheumatoid Arthritis-Associated Interstitial Lung Disease"[Title/Abstract] OR "RA-ILD"[Title/Abstract] OR "RA lung disease"[Title/Abstract] OR "arthritis associated interstitial lung disease"[Title/Abstract]) AND ("Lung Diseases, Interstitial"[MeSH] OR "Interstitial Lung Disease"[Title/Abstract]) AND ("Antifibrotic Agents"[MeSH] OR "nintedanib"[Title/Abstract] OR "pirfenidone"[Title/Abstract] OR "Antirheumatic Agents"[MeSH] OR "DMARDs"[Title/Abstract] OR "Glucocorticoids"[MeSH] OR "corticosteroids"[Title/Abstract] OR "Anti-Inflammatory Agents"[MeSH] OR "anti-inflammatory"[Title/Abstract] OR "anti-fibrotic"[Title/Abstract]) AND ("Therapeutics"[MeSH] OR "treatment"[Title/Abstract] OR "therapy"[Title/Abstract] OR "management"[Title/Abstract])) | January 1991 to August 2024 | 83,022 |
| **Embase** | 'Rheumatoid Arthritis-Associated Interstitial  Lung Disease' OR  'RA-ILD' OR  'arthritis-related interstitial lung disease' OR  'rheumatoid lung disease' OR  'interstitial lung disease' OR  'RA treatment' OR  'anti-fibrotic therapy' OR  'anti-inflammatory therapy' OR  'nintedanib' OR  'pirfenidone' OR  'DMARDs' OR  'corticosteroids' OR  'biologic DMARDs' | 1991 to 2024 | 1,194,681 |
| **Cochrane Library** | "Rheumatoid Arthritis" OR "lung disease" OR "interstitial lung disease" OR "pulmonary fibrosis" OR "arthritis related lung disease" OR "chronic lung disease" OR "respiratory disease" OR "treatment of lung disease" OR "anti-fibrotic therapy" OR "nintedanib" OR "pirfenidone" OR "anti-inflammatory" OR "corticosteroids" OR "disease modifying antirheumatic drugs" OR "biologic agents" OR "immunosuppressive therapy" OR "therapeutic management" OR "management of lung disease" | January 1991 to August 2024 | 88,243 |
| **Total** |  |  | **1,365,946** |
